# Evaluation of Mediators Associated with the Inflammatory Response in Prostate Cancer Patients Undergoing Radiotherapy

**DOI:** 10.1155/2018/9128128

**Published:** 2018-02-25

**Authors:** Nice Bedini, Alessandro Cicchetti, Federica Palorini, Tiziana Magnani, Valentina Zuco, Marzia Pennati, Elisa Campi, Paola Allavena, Samantha Pesce, Sergio Villa, Barbara Avuzzi, Sara Morlino, Maria Emanuela Visentin, Nadia Zaffaroni, Tiziana Rancati, Riccardo Valdagni

**Affiliations:** ^1^Radiation Oncology 1, Fondazione IRCCS Istituto Nazionale dei Tumori, Milan, Italy; ^2^Prostate Cancer Program, Fondazione IRCCS Istituto Nazionale dei Tumori, Milan, Italy; ^3^Department of Physics, Università degli Studi di Pavia, Pavia, Italy; ^4^Molecular Pharmacology, Fondazione IRCCS Istituto Nazionale dei Tumori, Milan, Italy; ^5^Laboratory of Cellular Immunology, Clinical and Research Institute Humanitas, Rozzano, Italy; ^6^Department of Oncology and Hemato-Oncology, Università degli Studi di Milano, Milan, Italy

## Abstract

A recent “hot topic” in prostate cancer radiotherapy is the observed association between acute/late rectal toxicity and the presence of abdominal surgery before radiotherapy. The exact mechanism is unclear. Our working hypothesis was that a previous surgery may influence plasma level of inflammatory molecules and this might result in enhanced radiosensitivity. We here present results on the feasibility of monitoring the expression of inflammatory molecules during radiotherapy. Plasma levels of a panel of soluble mediators associated with the inflammatory response were measured in prostate cancer patients undergoing radical radiotherapy. We measured 3 cytokines (IL-1b, IL-6, and TNF alpha), 2 chemokines (CCL2 and CXCL8), and the long pentraxin PTX3. 20 patients were enrolled in this feasibility evaluation. All patients were treated with IMRT at 78 Gy. 3/20 patients reported grade 2 acute rectal toxicity, while 4/20 were scored as grade 2 late toxicity. CCL2 was the most interesting marker showing significant increase during and after radiotherapy. CCL2 levels at radiotherapy end could be modelled using linear regression including basal CCL2, age, surgery, hypertension, and use of anticoagulants. The 4 patients with late toxicity had CCL2 values at radiotherapy end above the median value. This trial is registered with ISRCTN64979094.

## 1. Introduction

Prostate cancer is one of the most common tumours in the Western world. Radical prostatectomy and radiotherapy represent the two standard treatments. Both can be affected by significant side effects that can adversely condition patients' quality of life.

In the past decade, many advances have been made in terms of treatment outcomes and reduction of the side effects experienced by prostate cancer survivors. In the field of radiotherapy, this was primarily achieved through the introduction of sophisticated radiotherapy technologies. They allow the delivery of highly conformal doses to the tumor target through intensity modulated beams (IMRT), volumetric arcs ((volumetric modulated arc therapy (VMAT)), and precise image guidance (IGRT).

Nevertheless, a portion of the patient still suffers from radiation-induced toxicity and the availability of tools predicting unusual radiation toxicity could be crucial in improving the potential of individualizing the treatment with respect to several aspects, concerning the choice of the therapeutic strategy, dose prescription, fractionation, and use of supportive therapies.

A recent “hot topic” in prostate cancer radiotherapy is the observed association between acute/late rectal toxicity and the presence of abdominal surgery before radiotherapy [1, 2]. The reasons why surgery procedures not directly involving the irradiated volume may be so strongly correlated to late intestinal toxicity are still unknown and only some hypothesis can be suggested. It has been speculated that previous surgery may act throughout a limitation in blood supply and/or in reducing bowel movements [2].

The working hypothesis, which guided the present study, was that a previous surgery may influence plasma level of inflammatory molecules/cytokines, and this fact might result in an enhanced radiosensitivity. Surgery could function as a potential precursor of inflammatory patterns that could lead to an increased sensitivity even far from the surgical injury through cytokine-mediated reactions.

Cytokines are small proteins released by cells that act via receptors. The important role that cytokines play in mediating radiation toxicity was first reported by Rubin [3]; later, Okunieff [4] elucidated the link between inflammation, fibrosis, and tissue restitution. Some other investigators have even shown that the levels of inflammatory cytokines in individual animals of the same strain affects the severity of toxicity from animal to animal [5]. It is also well established that patients with intrinsically high inflammatory states (e.g., collagen vascular disease and autoimmune disease) are at extremely high risk of severe fibrosis after pelvis radiotherapy [6] and thus, we could expect that the variability of these cytokines among patients might explain the wide variability of clinical toxicity.

The role of these inflammatory molecules in the response of tissues to irradiation has also been related to the abscopal effect through adaptive immune responses [7].

The present analysis [8, 9] focused on three cytokines: interleukin 1 beta (IL-1b), interleukin 6 (IL-6), and tumor necrosis factor alpha (TNF alpha); two chemokines: chemokine ligand 2 (CCL2) and CXC chemokine ligand 8 (CXCL8); and the long pentatraxin, pentraxin 3 (PTX3) [10]. The primary aims were (a) to assess plasma levels of the selected inflammatory molecules in prostate cancer patients undergoing radical radiotherapy; (b) to study inflammatory molecule kinetics as a function of radiation dose and follow-up time; (c) to investigate the relationship between plasma levels of the selected inflammatory molecules and acute/late radiation-induced intestinal toxicity; and (d) to verify if abdominal surgery prior to radiotherapy influences the absolute plasma levels of inflammatory molecules and/or their kinetics.

## 2. Materials and Methods

### 2.1. Study Population

Twenty patients with a diagnosis of histologically confirmed, locally confined, prostate adenocarcinoma and receiving definitive intensity-modulated radiation therapy (IMRT) at 78 Gy (2 Gy/fraction) were enrolled in this pilot study. Six patients received neoadjuvant/adjuvant hormone therapy. Detailed characteristics of the patient population are given in Table 1. Patients were recruited from March 2011 to June 2012. This study was approved by the Fondazione IRCCS Istituto Nazionale dei Tumori Ethics Committee (INT 67/10), and written informed consent was obtained from all subjects prior to study enrolment.

### 2.2. Patient Blood Sampling, Processing, and Analysis

Ten millilitres of EDTA blood samples were obtained before radiotherapy (baseline), after a dose of 8 Gy, after 50 Gy, at radiotherapy end and one month after treatment completion. Samples were centrifuged for 20 minutes at 2200 r.c.f./4°C and immediately stored at ≤−80°C until analysis.

All analyses were carried out blind to patient and therapy factors. The amount of IL-1b, IL-6, CXCL8, TNF alpha, CCL2, and PTX3 was determined using commercially available ELISA kits (R&D Systems Inc., Minneapolis, MN, USA), according to the manufacturer's protocols.

### 2.3. Grading Radiation-Induced Acute Toxicity

Patients were examined at the start of treatment, once weekly during treatment, at the end of RT, and every six months thereafter till 5-year follow-up. Radio-induced was scored using a self-administered questionnaire. The questionnaire was previously used and validated in a pilot study with a subset of 50 patients enrolled within the retrospective study AIROPROS 0101 [11]. It consists of 10 questions, the answers to which are worded to be compatible with a 4-point categorical scale (1, not at all; 2, a little; 3, much; and 4, very much) which correspond to the SOMA/LENT (subjective objective management analytic/late effects on normal tissue) grading. With this questionnaire, four major types of rectal injury can be evaluated for rectal bleeding and mucosal loss, sphincter control and continence, stool frequency, and pain and urgency. English version of the questionnaire is reported in [12].

Acute rectal symptoms were defined as the maximum grade reached within one month after radiotherapy end. Late symptoms were determined as the maximum grade reached between 6 months and 5 years after treatment completion.

### 2.4. Statistical Analysis

All analyses were done using MedCalc (1993–2017 MedCalc Software bvba).

The Mann–Whitney *U* test was used to compare baseline/end of treatment plasma levels of the selected inflammatory molecules in patients with/without an abdominal surgery before radiotherapy.

Longitudinal evaluation of inflammatory molecule kinetics during radiotherapy and one month after treatment completion was analyzed in the frame of one-way analysis of variance (ANOVA) for multiple measures, in order to discover patterns of systematic variation with time.

## 3. Results

### 3.1. Results on Inflammatory Molecule Levels

**Figure 1:**
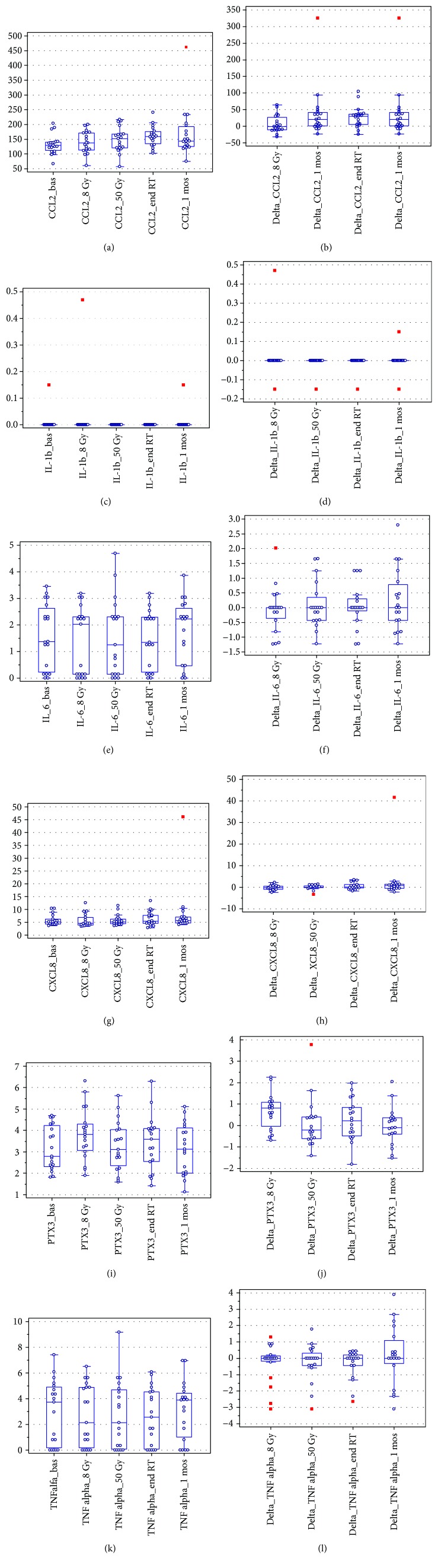
Plasma levels of the selected inflammatory molecules for all measurement points: absolute values (a, c, e, g, i, and k) and absolute variations with respect to baseline (b, d, f, h, j, and l). 1 mos: 1 month; RT: radiotherapy. Units of measure are pg/ml for IL-1b, IL-6, CXCL8, CCL2, and TNF alpha; ng/ml: PTX3.

Detailed descriptive results on plasma levels of the selected inflammatory molecules for all measurement points are given in Table 2 and Figure 1. IL-1b and IL-6 were fairly undetectable in most patients and presented with very low variability among patients.

TNF alpha levels were significantly different at baseline in patients with/without a previous abdominal surgery, median values 2.1 pg/ml versus 4.8 pg/ml, *p* = 0.05. CCL2 levels were lower in patients with surgery, both at baseline and radiotherapy end, but differences did not reach statistical significance (124.0 pg/ml versus 138.2 pg/ml, *p* = 0.22, and 150.5 pg/ml versus 168.2 pg/ml, *p* = 0.10, resp.).

Results of ANOVA for repeated measures are reported in Table 3. CCL2 was the most interesting marker, showing significant linear increase during and after radiotherapy. Median values were 127.4 pg/ml (baseline), 134.6 pg/ml (8 Gy), 145.8 pg/ml (50 Gy), 154.8 pg/ml (radiotherapy end), and 143.2 pg/ml (1 month after radiotherapy completion), *p* range: 0.01–0.05. PTX3 showed a quadratic trend, with a maximum at 8 Gy (*p* = 0.01), all other inflammatory markers did not exhibit systematic changes with radiotherapy dose/time.

**Figure 2:**
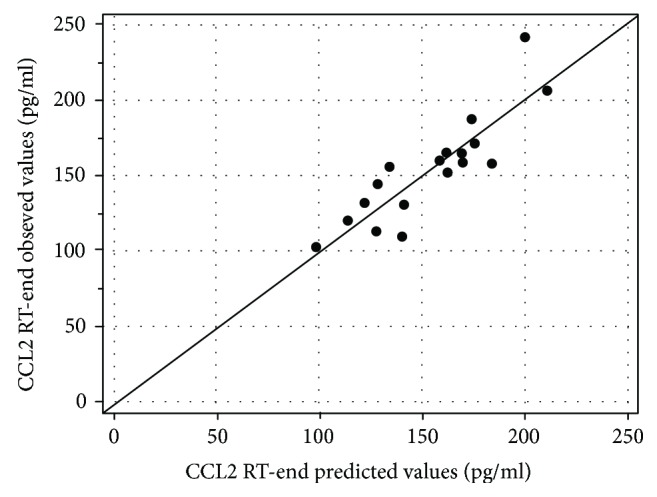
Calibration plot for the linear regression model predicting CCL2 levels at end of radiotherapy (observed CCL2 levels at radiotherapy end versus CCL2 values as predicted by the linear regression). Details are given in the text. Units of measure pg/ml. RT: radiotherapy.

CCL2 levels at radiotherapy end could be modelled using linear regression including the following variables: baseline CCL2 (coefficient = 1.15, *p* = 0.0001), age (coefficient = −3.26, *p* = 0.004), abdominal surgery (coefficient = 23.3, *p* = 0.09), hypertension (coefficient = 29.6, *p* = 0.02), and use of anticoagulants (coefficient = 41.0, *p* = 0.05) and multiple correlation coefficient = 0.89 (see plot). Significance level of analysis of variance for this linear regression was *p* = 0.002, multiple correlation coefficient was 0.87, and coefficient of determination *R*^2^ was 0.75.  Figure 2 shows the calibration plot (observed CCL2 levels at radiotherapy end versus CCL2 values as predicted by the linear regression model).

### 3.2. Results on Radio-Induced Toxicity

Three out of twenty patients (15%) reported grade 2 acute rectal toxicity, while 4/20 (20%) were scored as grade 2 late rectal toxicity in the first 36 months after radiotherapy completion. No grade 3-4 event was observed. Details on incidence of acute and late grade ≥ 1 toxicity (as determined by questions in the questionnaire) are reported in Table 4.

Multiple toxicity symptoms (>3) were experienced by 56.3% and 43.8% of patients in the acute and late phase, respectively.

The 4 patients with late toxicity had CCL2 values at radiotherapy end above the median value.

Detailed comparison of plasma levels for selected inflammatory markers for patients with/without multiple symptoms of acute/late intestinal toxicity is reported in Table 5. *t*-test was statistically different only for baseline TNF alpha: 2.0 versus 4.7 ng/ml, *p* = 0.04.

Incidence of acute fecal incontinence and rectal bleeding was slightly higher in the group of patients with previous abdominal surgery, 11% versus 0% and 33.3% versus 28.6% for incontinence and bleeding, respectively, differences were not statistically significant. Late rectal bleeding was also slightly higher for patients with previous abdominal surgery, 33.3% versus 28.6%, difference was not statistically significant. 67% of patients with abdominal surgery presented with at least three different acute intestinal toxicity symptoms versus 43% in the rest of the population; even in this case, difference was not statistically significant due to the very small size of the population of this pilot study.

## 4. Discussion

Preclinical and clinical studies have shown that radiotherapy induces cytokine responses that could play a major role in mediating radiation toxicity [3–6].

In the present study, grade ≥ 1 acute and late intestinal toxicity (as defined by questions in the patient-reported questionnaire) were not found to be significantly associated with plasma levels of inflammatory markers, with the only exception of baseline TNF alpha level, which was higher in patients experiencing multiple late intestinal toxicity symptoms. The small size of the study population could highlight only important differences, larger populations are required to investigate more modest variations.

When considering inflammatory molecule kinetics during and immediately after radiotherapy, CCL2 was found to significantly increase during IMRT. Though statistical association between moderate/severe radio-induced (acute and late) intestinal toxicity and CCL2 levels could not be investigated due to the low size of the study population, patients exhibiting late grade 2 toxicity were found to have CCL2 levels at the end of radiotherapy above the median value for the study cohort.

Interestingly, CCL2 levels at the end of radiotherapy could be modelled through linear regression including age, abdominal surgery, hypertension, and use of anticoagulants. All these features are known to be risk factors for increase intestinal toxicity after prostate cancer radiotherapy [13–15], thus suggesting a first possible link between patient clinical characteristics and his individual response in terms of biomarkers.

CCL2 is a low molecular weight monomeric polypeptide whose primary function was identified as promoting monocyte and macrophage migration to sites of inflammation [16]. For example, CCL2 is involved in monocyte infiltration in inflammatory diseases such as rheumatoid arthritis as well as in the inflammatory response against tumours.

There are limited data regarding the relationship between CCL2 and radiation exposure. Most results are related to the evidence that CCL2 overexpression in tumour is associated with macrophage infiltration and poor prognosis in human cancers and may play a pivotal role in creating the fertile environment in the bone for metastasis [17–19].

Connolly and coworkers [20] demonstrated that radiotherapy stimulates increased production CCL2 and CCL5 at the tumour site, while Kalbasi and colleagues [21] found that ablative radiotherapy for pancreatic ductal adenocarcinoma led to a significant increase in CCL2 production by tumour cells, with genetic deletion of CCL2 in pancreatic ductal adenocarcinoma cells improving radiotherapy efficacy.

When considering the association between CCL2 expression and response of normal tissues to radiation, only two studies are available in the literature and they reported interesting results. Lee et al. [22] showed that irradiation induces a transient nonclassical cytokine response with selective upregulation of CCL2. Interestingly, absence of CCL2 signalling in the hours after irradiation is sufficient to restore hippocampal neurogenesis and to decrease the risk of long-term defects in neural stem cell function following cranial radiation in children. Holler and coworkers [23] demonstrated that pravastatin has a mitigatory effect on radiation-induced vascular dysfunction in the skin in a mouse model. Remarkably, pravastatin limits the radio-induced increase of blood CCL2 expression and inflammatory cell migration in tissues.

When considering the other measured inflammatory molecules, IL-1b and IL-6 were fairly undetectable in most patients and presented with very low variability among patients: for these reasons, they were considered of no interest for the purpose of the present study. TNF alpha levels were found to be significantly different between patients with/without a previous abdominal surgery, but it did not exhibit significant changes as a function of radiation dose and did not result to be associated to acute/late toxicity. PTX3 showed a quadratic trend with an early increase with dose (at 8 Gy) with subsequent return to baseline levels by the end of treatment. Increase in PTX3 was not associated with patient-reported morbidity.

To our knowledge, there is only one previously published study investigating cytokine expression during IMRT for prostate cancer and their relationship with acute toxicity [24]. Their study population consisted of 22 prostate patients treated with exclusive IMRT (78 Gy at 2 Gy/fraction) and 20 patients receiving radiotherapy after prostatectomy (70 Gy at 2 Gy/fraction). They found IL-6 levels to be significantly elevated over baseline in the postprostatectomy group but no significant difference in the exclusive IMRT cohort. Increases in IL-2 and IL-1 levels over baseline were significantly associated with increased gastrointestinal and genitourinary toxicity, respectively, regardless of the radiotherapy regimen (exclusive IMRT versus postprostatectomy IMRT) regimen, while the analysis of IL-6 suggested that the increase of IL-6 was associated with a higher risk for gastrointestinal toxicity but it did not reach statistical significance.

Presence of previous abdominal surgery was not found to be significantly associated with toxicity or to plasma levels of inflammation markers in this pilot study. As already pointed out, interestingly, previous abdominal surgery was included as a factor modulating CCL2 levels at radiotherapy end, together with other patient features known to be predictors of intestinal toxicity. This modulating effect should be confirmed on a wider population, in order to suggest a direct effect of factors associated with toxicity on CCL2 levels at radiotherapy end.

Besides interest in the comprehension of the biological rationale for the correlation of some clinical factors with morbidity, investigation of association between inflammatory molecule levels and radio-induced toxicity is of interest because it could have the potential of being a biological measure of the individual patient radiosensitivity, thus prompting further optimization of radiotherapy treatment for more sensible patients or dose escalation on resistant patients. Prophylactic treatment of toxicity symptoms could also be proposed in patients at higher risk of enhanced inflammation processes.

One important limitation of this study is related to the limited sample size. Our pilot study was intended to be exploratory, to inspect the feasibility of multiple blood samples for biomarker investigation in the frame of clinical practice and to validate the study methodology. These results are expected to guide future, larger trials which could establish the time course of plasma levels of inflammatory molecules after radiotherapy and how they are associated with normal tissue radiation toxicity. Specific future research topics should include evaluation of a wider spectrum of radio-induced symptoms (e.g., including urinary and hematologic toxicities) and of populations of patients treated with radiotherapy for different cancer types, such as head-and-neck patients or breast cancer patients. Interaction with concomitant oncological treatment (such as chemotherapy or hormone therapy) should be considered.

## 5. Conclusions

This preliminary study identified a correlation between CCL2 levels at the end of radiotherapy and basal CCL2, age, and surgery, suggesting a different response to radiotherapy in older patients and in patients with pretreatment abdominal surgery. Interestingly, these clinical characteristics are the same features included in predictive models for acute and late rectal toxicity. Larger accrual is needed to confirm these feasibility results and to study the association with radio-induced acute and late toxicity.

## Figures and Tables

**Table 1:** Details on study population characteristics.

	Variable^
Age	71 yrs (53–78 yrs)
PSA at diagnosis	7 ng/ml (2.5–14.8)
Clinical stage	13 cT1
	5 cT2
	2 cT3
Gleason pattern score	13 GPS = 3 + 3
	7 GPS = 3 + 4
Neoadjuvant/adjuvant hormone therapy	6
BMI	25 (24–32)
Diabetes	1
Hypertension	13
Previous abdominal surgery	12
Use of anticoagulants	2

^Median value is reported together with range for continuous variable and prevalence of patients with the selected feature for dichotomic/categorical variables. PSA: prostate-specific antigen; BMI: body mass index; yrs: years.

**Table 2:** Detailed descriptive results on plasma levels of the selected inflammatory molecules for all measurement points.

pg/ml or ng/ml^	Mean	SD	Median	Minimum	Maximum	75th perc	25th perc
CCL2 baseline	131.5	32.1	127.4	66.7	203.3	110.0	139.8
CCL2 8 Gy	136.8	37.7	134.6	60.5	200.4	111.9	171.4
CCL2 50 Gy	146.6	41.8	145.8	57.7	217.1	118.6	167.1
CCL2 end RT	157.3	34.5	158.4	102.6	240.8	131.2	174.1
CCL2 1 mos	167.9	79.7	143.2	74.6	461.7	127.5	187.2

IL-1b baseline	0.0	0.0	0.0	0.0	0.2	0.0	0.0
IL-1b 8 Gy	0.0	0.1	0.0	0.0	0.5	0.0	0.0
IL-1b 50 Gy	0.0	0.0	0.0	0.0	0.0	0.0	0.0
IL-1b end RT	0.0	0.0	0.0	0.0	0.0	0.0	0.0
IL-1b 1 mos	0.0	0.0	0.0	0.0	0.2	0.0	0.0

IL-6 baseline	1.6	1.2	1.6	0.0	3.5	0.3	2.5
IL-6 8 Gy	1.4	1.3	2.1	0.0	3.2	0.2	2.3
IL-6 50 Gy	1.6	1.4	1.6	0.0	4.7	0.2	2.3
IL-6 end RT	1.6	1.1	1.8	0.0	3.2	1.3	2.3
IL-6 1 mos	1.7	1.2	2.2	0.0	3.9	0.5	2.5

CXCL8 baseline	5.7	2.0	5.2	3.7	10.4	4.3	5.9
CXCL8 8 Gy	5.5	2.4	4.6	3.4	12.5	3.9	6.4
CXCL8 50 Gy	5.7	2.0	5.1	3.7	11.4	4.4	6.2
CXCL8 end RT	6.3	2.7	5.4	2.9	13.4	4.6	7.7
CXCL8 1 mos	8.3	9.1	5.7	4.1	46.1	4.7	7.2

PTX3 baseline	3.3	1.1	3.0	1.8	5.6	2.3	4.3
PTX3 8 Gy	3.9	1.2	3.9	1.9	6.3	3.1	4.6
PTX3 50 Gy	3.3	1.1	3.3	1.6	5.6	2.5	4.0
PTX3 end RT	3.5	1.3	3.7	1.4	6.3	2.6	4.1
PTX3 1 mos	3.1	1.1	3.1	1.1	5.1	2.0	4.0

TNF alpha baseline	3.1	2.5	3.9	0.0	7.4	0.4	4.9
TNF alpha 8 Gy	2.7	2.3	2.9	0.0	6.5	0.4	4.9
TNF alpha 50 Gy	2.8	2.6	2.7	0.0	9.2	0.2	4.6
TNF alpha end RT	2.7	2.2	2.8	0.0	6.1	0.2	4.5
TNF alpha 1 mos	3.1	2.2	3.6	0.0	7.0	1.2	4.3

^pg/ml for IL-1b, IL-6, CXCL8, CCL2, and TNF alpha; ng/ml: PTX3; SD: standard deviation; perc: percentile; 1 mos: 1 month; RT: radiotherapy.

**Table 3:** Results of one-way analysis of variance for repeated measures for all considered markers. Best trend for marker values as a function of time is reported.

	Best trend	*p* value
IL-1b	Cubic	0.34
IL-6	Quadratic	0.16
CXCL8	Linear	0.19
CCL2	Linear	0.01^∗^
TNF alpha	Quadratic	0.07
PTX3	Quadratic	0.06

^∗^Statistically significant.

**Table 4:** Details on incidence of acute and late grade ≥ 1 toxicity (as determined by questions in the questionnaire).

	Acute incidence grade ≥ 1	Late incidence grade ≥ 1
Stool frequency	50.0%	50.0%
Diarrhea	62.5%	31.3%
Tenesmus	43.8%	25.0%
Stool urgency	31.3%	43.8%
Fecal incontinence	6.3%	6.3%
Rectal pain	18.8%	25.0%
Rectal bleeding	31.3%	31.3%

**Table 5:** Comparison of plasma levels for the selected inflammatory molecules in subgroups of patients experiencing multiple symptoms (≥3) for intestinal acute/late toxicity. *p* values for test are reported.

	Acute toxicity						Late toxicity					
	Patients with <3 symptoms		Patients with ≥3 symptoms		*t*-test		Patients with <3 symptoms		Patients with ≥3 symptoms		*t*-test	
	Mean	SD	Mean	SD	Mean diff	*p*	Mean	SD	Mean	SD	Mean diff	*p*
CCL2 baseline	139.4	34.1	134.2	30.4	−5.2	0.75	145.5	37.3	124.9	16.5	−20.7	0.18
CCL2 50 Gy	155.7	33.9	159.7	40.9	4.1	0.83	162.4	38.5	152.3	36.7	−10.1	0.60
CCL2 end RT	161.2	20.7	165.8	42.4	4.6	0.79	165.5	39.1	161.6	28.1	−3.9	0.82
CCL2 1 mos	153.2	40.0	201.3	104.2	48.1	0.25	193.9	107.4	162.6	39.6	−31.3	0.46
IL-8 baseline	6.9	2.7	5.2	1.5	−1.7	0.15	6.4	2.6	5.4	1.6	−0.9	0.40
IL-8 50 Gy	6.6	2.5	5.4	1.9	−1.2	0.29	6.2	2.4	5.6	2.0	−0.6	0.60
IL-8 end RT	8.2	3.2	5.4	2.1	−2.8	0.06	7.6	3.1	5.4	2.3	−2.2	0.13
IL-8 1 mos	7.1	2.2	10.6	13.4	3.4	0.50	11.6	13.0	5.8	2.1	−5.8	0.25
PTX3 baseline	3.1	1.2	3.2	1.1	0.2	0.75	2.9	1.1	3.5	1.0	0.5	0.37
PTX3 50 Gy	3.3	1.5	3.1	1.1	−0.2	0.72	3.3	1.4	3.0	1.1	−0.3	0.69
PTX3 end RT	3.2	1.4	3.4	1.4	0.2	0.80	3.6	1.7	3.0	0.8	−0.6	0.40
PTX3 1 mos	3.2	1.3	3.0	1.2	−0.2	0.72	3.1	1.2	3.1	1.3	0.0	0.98
TNF alpha baseline	2.0	2.3	4.0	2.6	2.0	0.13	*2.0*	*2.2*	*4.7*	*2.3*	*2.7*	*0.04*
TNF alpha 50 Gy	2.1	2.5	3.6	3.0	1.5	0.29	2.0	2.4	4.0	3.1	2.0	0.17
TNF alpha end RT	1.9	2.3	3.3	2.4	1.3	0.27	1.9	2.2	3.7	2.3	1.8	0.14
TNF alpha 1 mos	2.3	2.3	3.8	2.3	1.5	0.23	2.5	2.6	4.1	1.8	1.6	0.17

^pg/ml for IL-1b, IL-6, CXCL8, CCL2, and TNF alpha; ng/ml: PTX3; SD: standard deviation; 1 mos: 1 month; RT: radiotherapy; diff: difference.
